# Cerebral vascular control is associated with skeletal muscle pH in chronic fatigue syndrome patients both at rest and during dynamic stimulation^☆^

**DOI:** 10.1016/j.nicl.2012.12.006

**Published:** 2013-01-05

**Authors:** Jiabao He, Kieren G. Hollingsworth, Julia L. Newton, Andrew M. Blamire

**Affiliations:** a Institute of Cellular Medicine & Newcastle Magnetic Resonance Centre, Newcastle University, Newcastle upon Tyne, United Kingdom; b Institute for Ageing and Health, Newcastle University, Newcastle upon Tyne, United Kingdom

**Keywords:** Autonomic function, Chronic fatigue syndrome, ^31^P MR spectroscopy, Cerebral blood flow, Arterial spin labelling (ASL), Dual echo fMRI

## Abstract

Cerebral blood flow (CBF) is maintained despite changing systemic blood pressure through cerebral vascular control, with such tight regulation believed to be under local tissue control. Chronic fatigue syndrome (CFS) associates with a wide range of symptoms, including orthostatic intolerance, skeletal muscle pH abnormalities and cognitive impairment. CFS patients are known to have reduced CBF and orthostatic intolerance associates with abnormal vascular regulation, while skeletal muscle pH abnormalities associate with autonomic dysfunction. These findings point to autonomic dysfunction as the central feature of CFS, and cerebral vascular control being influenced by factors outside of the brain, a macroscopic force affecting the stability of regional regulation. We therefore explored whether there was a physiological link between cerebral vascular control and skeletal muscle pH management in CFS.

Seventeen consecutive CFS patients fulfilling the Fukuda criteria were recruited from our local CFS clinical service. To probe the static scenario, CBF and skeletal muscle pH were measured at rest using MRI and ^31^P magnetic resonance spectroscopy (^31^P-MRS).

To examine dynamic control, brain functional MRI was performed concurrently with Valsalva manoeuvre (VM), a standard autonomic function challenge, while ^31^P-MRS was performed during plantar flexion exercise.

Significant inverse correlation was seen between CBF and skeletal muscle pH at rest (r = − 0.67, p < 0.01). Prolonged cerebral vascular constriction during the sympathetic phase of VM was associated with higher pH in skeletal muscle after plantar flexion exercise (r = 0.69, p < 0.008).

In conclusion, cerebral vascular control is closely related to skeletal muscle pH both at rest and after dynamic stimulation in CFS.

## Highlights

► Cerebral vascular control in CFS is affected by factors external to the brain. ► Valsalva manoeuvre performed during fMRI probes cerebral vascular control. ► Cerebral blood flow is associated with skeletal muscle pH at rest in CFS. ► Cerebral vascular control relates to skeletal pH recovery after stimulation in CFS. ► Our results point towards a peripheral or systemic cause for CFS.

## 1 Introduction

Cerebral blood flow (CBF) is maintained at a constant level across a range of blood pressure (BP) (Lassen, 1964). Although the cerebral vascular control mechanism is local in healthy subjects, it is affected by conditions such as hypertension (Immink et al., 2004) and chronic fatigue syndrome (CFS) (Sutcliffe et al., 2010).

Although CFS (Evengard et al., 1999; Sanders and Korf, 2008) is classified as a nervous system disease, its cause is unknown, and a number of factors have been shown to be related to the disease progression (Afari and Buchwald, 2003). Almost 90% of CFS patients describe orthostatic symptoms (Newton et al., 2007) and autonomic dysfunction is a frequent finding (Hollingsworth et al., 2010a). CFS patients have reduced CBF (Biswal et al., 2011) and orthostatic intolerance is associated with prolonged cerebral vascular constriction after autonomic challenge (Lin et al., 2011). In our own studies, we have shown effects in peripheral tissue including compromised skeletal muscle response to exercise, with CFS patients generating higher levels of acid within their muscle compared to matched sedentary controls (Hollingsworth et al., 2010a; Jones et al., 2010). We have also already confirmed that those CFS patients with the skeletal muscle abnormality were significantly more likely to have concurrent impaired cardiac energetics (Hollingsworth et al., 2012) and that the impairment of skeletal muscle pH handling correlates with the autonomic dysfunction (Jones et al., 2010). In combination, this data suggests that autonomic dysfunction is a central feature of CFS, indictable by pH handling measurement in skeletal muscle.

Given its local nature, we hypothesise that cerebral vascular control in CFS is affected by autonomic dysfunction, and this relationship would manifest itself in a correlation between the pH handling in skeletal muscle and cerebral vascular control.

To test our hypothesis in a resting state we performed arterial spin labelling (ASL) magnetic resonance imaging (MRI) to measure CBF in the brain and used ^31^P magnetic resonance spectroscopy (MRS) to probe skeletal muscle pH.

**Fig. 1:**
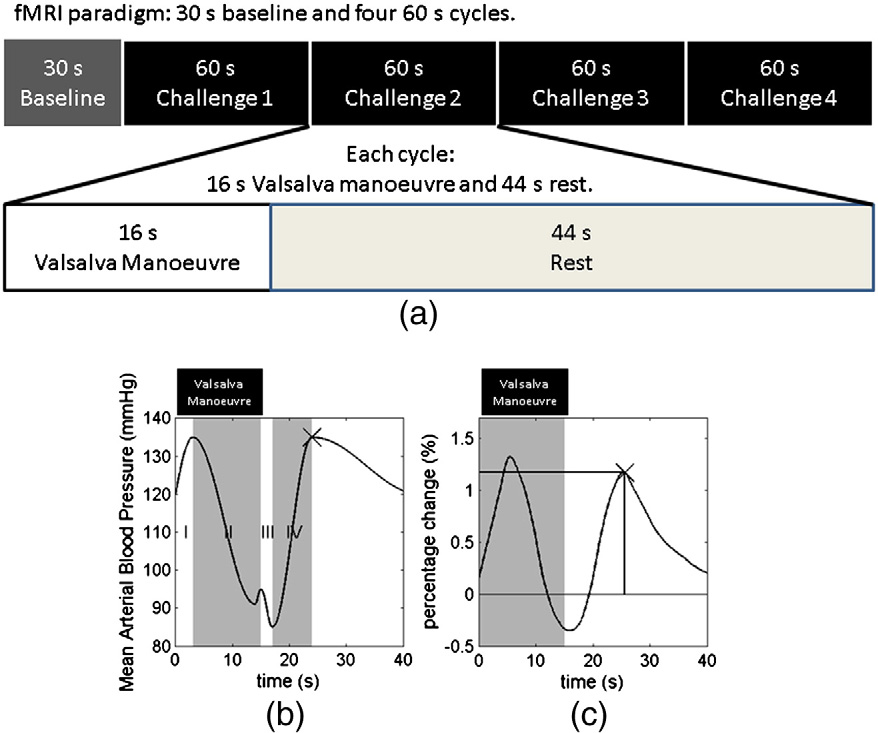
Shows (a) the schematic paradigm of the fMRI experiment, (b) the schematic diagram illustrating the mean arterial blood pressure (MABP) time course during the four phases of Valsalva manoeuvre, and (c) the group mean of cycle averaged time course measured in fMRI experiment. The fMRI paradigm was composed of 30 s baseline and four 1 min cycles. Each cycle was composed of 16 s Valsalva manoeuvre and 44 s rest. The BP response is divided into four phases, each labelled in different shades and marked as “I”, “II”, “III” and “IV”. The characteristic peak associated with sympathetic function is marked by “x”. The fMRI protocol monitors a signal related to cerebral vascular dilation. Individual time courses were normalised to their respective baseline signal level to derive the percentage change time course. The onset of the Valsalva manoeuvre is aligned to 0 s, while its duration is marked by the grey window. For each time course, the peak associated with sympathetic function, marked by “x”, was identified, with its time (dotted line) and magnitude (solid line) measured.

The Valsalva manoeuvre (VM) has been widely used to identify deficits in autonomic and cardiac function (Remmen et al., 2006; Woo et al., 2007) as it induces phased changes in BP (Dawson et al., 1999) (Fig. 1b). In particular, sympathetic function is characterised by a rapid increase in BP at the end of the VM (marked “x” in Fig. 1b). The vascular dilation associated with sympathetic function can be detected by functional MRI (fMRI), tailored to reflect transient changes in blood volume (Glover et al., 1996). Plantar flexion exercise demonstrated a skeletal muscle pH handling abnormality in CFS patients (Jones et al., 2010). The recovery of pH in skeletal muscle after exercise is assisted by modulated blood flow through changing vascular calibre, a factor influenced by the autonomic function.

To examine the relationship between cerebral vascular control and skeletal muscle pH handling in response to dynamic stimulation, we explored the relationship between cerebral vascular parameters during the VM through fMRI and skeletal muscle pH during plantar flexion exercise through ^31^P MRS.

## 2 Methods

Seventeen consecutive CFS patients were recruited from the local CFS clinical service based at the Newcastle upon Tyne Hospitals NHS Foundation Trust. All participants fulfilled the CDC 1994 (Fukuda) diagnostic criteria for CFS (Fukuda et al., 1994). The study was reviewed and approved by the Newcastle and North Tyneside Local Ethics Committee. The sponsor was Newcastle upon Tyne Hospitals NHS Foundation Trust and all participants provided written informed consent prior to the experiment. The study was performed on a 3 T whole body MR scanner (Achieva, Philips Medical Systems, The Netherlands). Two separate appointments were made for each patient to look at the brain and skeletal muscle, with a median time gap of 18 days.

### 2.1 Brain imaging

All the brain scans were performed in a single session for each patient using an 8 channel SENSE coil. Scans included 3D T_1_ weighted anatomical images, resting CBF maps and dynamic imaging of the VM in a functional MRI study.

#### 2.1.1 Anatomical images

Anatomical images in sagittal orientation were acquired using a standard T_1_ weighted clinical protocol with a resolution of 1 × 1 × 1.2 mm^3^, field of view (FOV) of 240 × 240 × 216 mm^3^, repetition time (TR) of 8.1 ms and echo time (TE) of 4.6 ms. Segmentation in SPM8 (Frackowiak, 2004) was performed on the anatomical images to generate patient specific grey matter masks.

#### 2.1.2 Resting CBF mapping

Resting CBF was measured using an arterial spin labelling (ASL) based sequence (Kim, 1995; He and Blamire, 2010), with spiral readout module, TE of 11.13 ms, TR of 4 s, 4 × 4 mm^2^ in-plane resolution, FOV of 256 × 256 mm^2^, 30 averages and inflow time of 1500 ms. The image volume covered 14 contiguous slices of 6 mm thickness, which was positioned parallel to the anterior commissure (AC)–posterior commissure (PC) line and centred at the corpus callosum. Images were processed in SPM8 to correct for patient movement (Frackowiak, 2004). The grey matter mask, generated from the anatomical images, was applied to the perfusion weighted images, and subsequently the grey matter CBF was quantified (Kim and Tsekos, 1997).

#### 2.1.3 Functional MRI

To investigate the effect of the Valsalva manoeuvre (VM) on the cerebral circulation, subjects underwent a stimulus paradigm consisting of a 30 s baseline period followed by four 1 min cycles each comprising of a 16 s duration VM followed by a 44 s rest period (Fig. 1a). The physiological responses (Fig. 1b) to the VM have four phases (Dawson et al., 1999; Stolz et al., 2010): (I) onset of the initial strain increases intrathoracic pressure and pulmonary blood is forced into the left atrium to increase cardiac stroke volume and mean arterial blood pressure (MABP); (II) due to reduced venous return, the stroke volume decreases causing transient reduction in MABP until sympathetic activation induces vascular constriction causing MABP and heart rate to rise; (III) immediately after releasing the breathing pressure, the reduced pressure in the chest induces a reduction in stroke volume and further vascular constriction; (IV) with unimpeded venous return, cardiac output increases rapidly and rises above normal levels, before returning to the resting condition under the sympathetic control. Functional MRI used a dual echo time (TE) gradient echo EPI sequence to characterise the brain response to VM. An imaging volume was selected parallel to the AC–PC line and centred at the anterior part of the corpus callosum (20 slices, 4 mm thickness, 2.1 × 2.1 mm^2^ in-plane resolution, 112 × 112 matrix size, TR of 2 s, TE of 14.0 ms and 39.2 ms). This method provided two levels of sensitivity to the cerebrovascular response, with the short echo time signal considered in this report being mainly sensitive to the tissue water density (data from the second echo not reported here). Transient changes in this signal during stimulation reflect the vascular dilation (Glover et al., 1996). An air pressure monitoring system was employed to measure performance of the VM, consisting of a mouthpiece (POWERbreath, HaB direct, UK) adapted with a 30 ml Luer lock syringe and anti bacterial air filter (Albert Waeschle, UK) and connected via oxygen tubing to a purpose built electronic air pressure meter. Instructions to begin and end each VM were visually presented to the subject in the scanner via a projection system. During the VM, subjects were instructed to maintain an exhaled air pressure of 40 mm Hg and were presented with real-time pressure feedback which was marked out as a target line on the visual display. Functional MRI time series data were processed in SPM8 to extract the time course of the signal changes. Within the dataset for each subject, all scans were aligned, spatially smoothed using an 8 mm full width at half maximum (FWHM) Gaussian kernel and co-registered with anatomical images using default configurations (Frackowiak, 2004). The grey matter mask, generated from the anatomical images, was then applied as a global region of interest and the average time courses extracted. Each time course was subsequently smoothed and interpolated to 0.1 s temporal resolution using a spline algorithm (De Boor, 1978). Average fMRI responses to the VM were created for each individual subject. To correct for response variations, the onset of each individual VM was defined as the time point at which the air pressure reached 5 mm Hg (based on the recorded air pressure trace). A time course of 40 s duration was extracted for each cycle, starting at the detected onset time and the 4 responses averaged. The cycle averaged time courses were then normalised to the baseline signal level to obtain percentage change time courses. The timing and magnitude of the recovery peak were measured (marked by ‘x’ in Fig. 1c).

### 2.2 Muscle MR spectroscopy

**Fig. 2:**
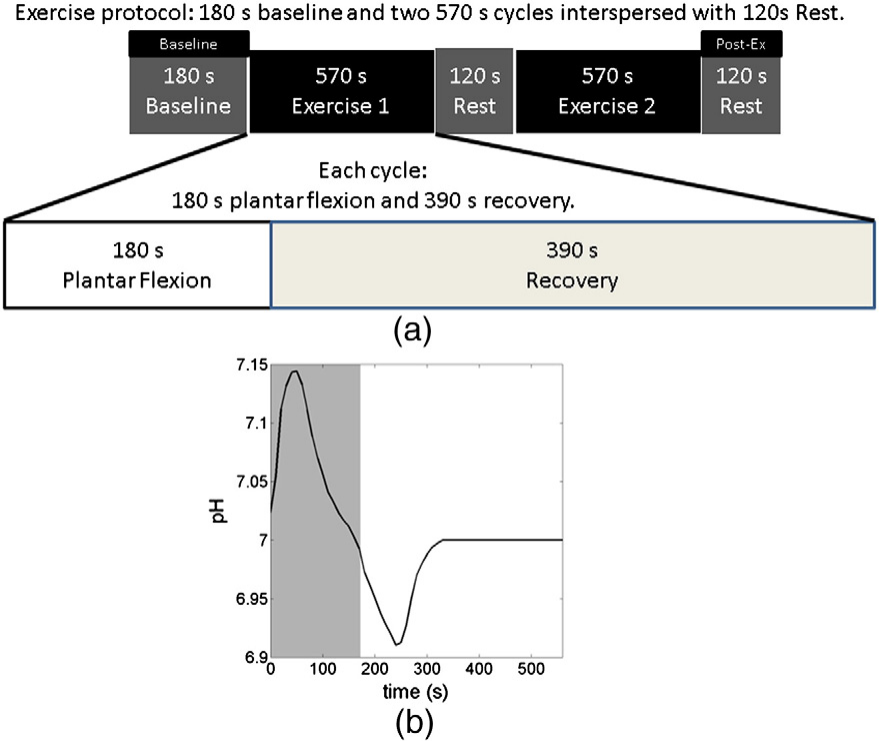
Shows (a) the schematic protocol for skeletal muscle exercise, and (b) a schematic pH response illustration during a plantar flexion cycle. The skeletal muscle exercise protocol contained 2 min baseline and three 9.5 min cycles interspersed by 2 min rest. Each cycle consisted of 3 min plantar flexion exercise and 6.5 min recovery period. The resting pH was measured during the baseline period, marked as “Baseline” in the figure. The recovered pH was measured during the rest period, marked as “Post-Ex” in the figure, after two plantar flexion cycles to minimise muscular adaptation effects. The period of plantar flexion exercise is marked by the grey window on the pH time course.

MRS data acquisition was performed on a different day to brain imaging, to avoid potential physiological interference between the Valsalva manoeuvre and skeletal muscle exercise. MRS data acquisition and quantification (Kemp and Radda, 1994; Kemp et al., 1997; Vanhamme et al., 1999) were performed in the same manner as our previous work (Hollingsworth et al., 2008). The exercise protocol (Fig. 2a) comprised a 180 s baseline followed by two 570 s long exercise cycles interspersed by a 120 s rest period. Each exercise cycle contained 180 s plantar flexion with a fixed load of 35% of the maximum voluntary contraction (Hollingsworth et al., 2008) and a 390 s recovery period. Resting muscle pH was measured from spectra acquired during the pre-exercise baseline period (marked as “Baseline” in Fig. 2a). Post exercise recovered pH was measured from the second plantar flexion cycle (marked as “Post-Ex” in Fig. 2a), with the first plantar flexion cycle performed to remove muscular adaptation effects (Hollingsworth et al., 2010b; Jones et al., 2012).

### 2.3 Statistical analysis

Statistical analysis was performed using SPSS statistical software (Version 19, IBM, New York, USA). To examine the relationship between baseline CBF and pH in the skeletal muscle at rest, linear regression (comparison 1) was performed on the CBF and resting pH. To investigate the relationship between cerebral vascular dilation and pH in skeletal muscle after physical challenge, the time and magnitude of the fMRI characteristic peak (marked out on Fig. 1c) were linearly regressed against the recovered pH measured after plantar flexion cycles (comparisons 2 and 3).

## 3 Results

The dataset showing the mean fMRI signals averaged across the subjects is shown in Fig. 1c and shows the multiple phases seen in the expected BP response during the performance of the VM.

**Fig. 3:**
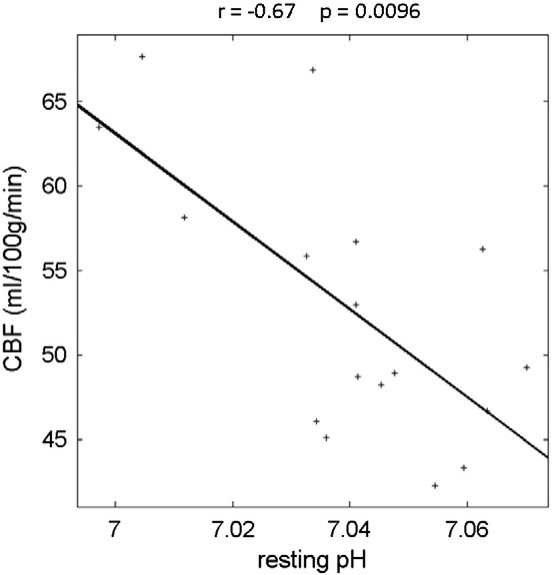
Shows that there is a significant correlation between cerebral blood flow (CBF) measured in brain and the resting pH measured during baseline in skeletal muscle. The strong negative correlation indicates that in CFS patients higher alkalinity in skeletal muscle is associated with lower CBF, while higher acidity is associated with higher CBF.

Resting cerebral blood flow (CBF) in the CFS patients was significantly inversely correlated with skeletal muscle resting pH measured during the baseline period (Fig. 3, r = − 0.67, corrected p < 0.01) indicating that a higher acidity within the skeletal muscle at rest was associated with increased CBF.

**Fig. 4:**
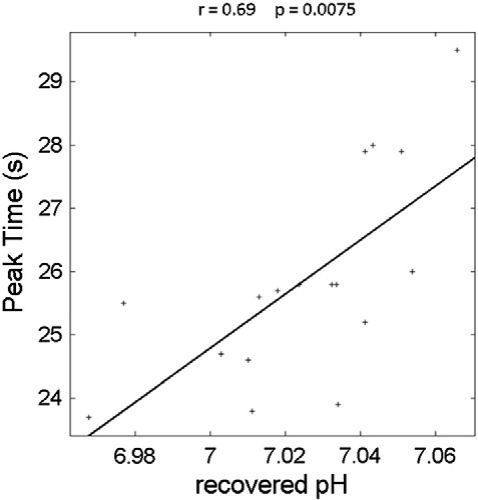
Shows the significant correlation between the cerebral vascular dilation characteristic peak time and the recovered pH measured during the rest between plantar cycles (shown in Fig. 2). The positive correlation indicates that an earlier cerebral vascular dilation peak related to sympathetic function is associated with a lower recovered pH.

**Fig. 5:**
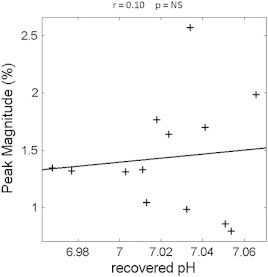
Shows the non-significant relationship between the cerebral vascular dilation characteristic peak magnitude and the recovered pH measured during the rest between plantar cycles (shown in Fig. 2).

When we explored the responses during the VM, there was also significant correlation (Fig. 4, r = 0.69, corrected p < 0.008) between the vascular dilation sympathetic peak time (marked by ‘x’ in Fig. 1c, corresponding to the time of maximal vascular dilation after VM), and the recovered pH (marked as ‘Post-Ex’ Fig. 2a), measured after a fixed period of recovery at the end of plantar flexion exercise; this positive correlation indicates that a delayed vascular dilation peak is associated with a higher (more alkaline) recovered pH. There was no significant correlation between the magnitude of the sympathetic peak and recovered pH (Fig. 5, r = 0.10, corrected p = NS), indicating that the vascular dilation in sympathetic phase of the VM is not significantly related to recovered skeletal muscle pH.

## 4 Discussion

This study has found that cerebral vascular control and skeletal muscle pH regulation are closely related, both at rest and when responding to dynamic stimulation in CFS patients, pointing to cerebral vascular control being affected by autonomic dysfunction.

CFS patients have been shown to have reduced CBF (Biswal et al., 2011) and higher skeletal muscle pH at rest (Jones et al., 2010). In this study we have shown a significant correlation (Fig. 3) between these parameters when measured in the same subject.

Since the vascular constriction and dilation are distinctively associated with each phase of the physiological responses in VM, the vascular dilation time course measured in the brain using fMRI allows the identification of the four phases. The vascular dilation characteristic peak is at the end of phase IV, where vascular dilation and sympathetic control reach their maximum. We have shown that CFS patients have higher recovered skeletal muscle pH (more alkaline) (Jones et al., 2012); and it has been shown that orthostatic intolerance is associated with prolonged vascular constriction after autonomic challenge (Lin et al., 2011). A significant correlation (Fig. 4) was found between the recovered pH and the vascular dilation characteristic peak time.

Although conventionally CFS has been considered to be a disease with primary CNS pathologies and secondary peripheral consequences, our results point to possible alternative disease mechanisms. It is possible that CFS is driven by a primary *peripheral* abnormality that is associated with secondary central sequelae, where a compromised skeletal muscle cellular membrane function underpins the observed abnormalities. The blood pH is generally maintained in a very narrow range through gas composition. Only very limited clinical conditions manifest themselves through altered blood pH. It has been shown that CFS patients often experience hyperventilation (Bogaerts et al., 2007), indicating acidic pressure on blood pH, since blood pH modulates breathing activity. However, hyperventilation also increases the concentration of oxygen in the blood, resulting in a vasoconstrictive effect, in turn reducing CBF at rest and which would prolong cerebral vascular constriction after autonomic challenge. The ^31^P MRS measures the intracellular pH, where CFS patients showed higher pH (more alkaline) both at rest (Jones et al., 2010) and after a fixed recovery period (Jones et al., 2012). The pH in skeletal muscle is tightly regulated to maintain enzyme functions, while blood has higher pH (more alkaline). We postulate that a compromised skeletal muscle cellular membrane function may lead to the equalisation of the pH between the skeletal muscle intracellular environment and blood, where an increase in intracellular pH (more alkaline) and decrease in blood pH (acidosis) take place, triggering hyperventilation to buffer the pH change in the blood. Furthermore, we have also shown that the nadir pH (Fig. 2b) during plantar flexion cycle is reduced (more acidic) in CFS patients, while its recovery to baseline is slower (Jones et al., 2012). This is in agreement with the suggestion that increased oxygen concentration in the blood (hyperoxia) compromises the vasodilation, aggravating acid accumulation and reducing the ability of waste removal. It has also been shown that plasma lipid peroxidation is elevated in CFS patients (Brkic et al., 2010), indicating a poor membrane integrity. It has also been shown that supplements of essential fatty acids can relieve the symptoms in CFS patients (Warren et al., 1999), while antioxidant supplements have been shown to be effective in the animal model (Singh et al., 2002). However, blood acidosis may also affect cellular membrane function, hence the underpinning mechanism of CFS cannot be fully resolved directly in this work, and extensive further work with detailed blood composition analysis is necessary to validate the pathophysiological model implicated here. Nevertheless, our results point to a disease mechanism outside of the CNS, with a peripheral cause.

Despite the current lack of consensus as to the underlying biological basis of CFS, there is considerable evidence, we believe supported by the current study, to highlight an abnormality of the autonomic nervous system as a unifying pathological factor. However, studies have suggested that in those with CFS there are other established aspects of this illness, including HPA-axis abnormalities (Cleare et al., 2001), central sensitization (Meeus and Nijs, 2007) and cognitive problems (Beaumont et al., 2012). Our findings of the changes in vascular control could provide the underpinning abnormality that explains these apparently disparate problems experienced by those with CFS.

There is no control group in this study. However, there is a wealth of literature showing that CFS patients have abnormal vascular control, as well as abnormal skeletal muscle pH management. The purpose of this study was to examine whether these two abnormalities are related to each other. Since these abnormalities are well known and documented, the absence of a control group does not affect the conclusions of this study. Despite the fact that the number of patients enrolled in this study was not large, the strength of the correlations presented in this work is high, indicating the close relationship between the examined parameters. CFS is a heterogeneous disorder in terms of pathophysiology. Our results provide initial evidence that there is a commonality in terms of the characteristics of CFS. Despite heterogeneity, it is recognised that CFS patients often suffer from autonomic dysfunction and skeletal muscle pH abnormality. Our results show that these common abnormalities in CFS are closely linked in the patient cohort studied. However, larger scale studies should be commenced to examine the effects of heterogeneity on the observed relationships. Although the results presented in this work, in conjunction with literature findings, point towards alternative disease mechanisms, the results are correlative and do not prove a causal relationship. These drawbacks warrant further investigations on the direction initiated by this work.

This study is not only informative from scientific point of view, but also provides foundation for clinical management of CFS. The VM is a standard autonomic function challenge inducing large variations in systemic BP. Since CFS patients often suffer from autonomic function abnormalities and orthostatic intolerance in daily life, the VM provides a useful tool to explore the effects of autonomic/orthostatic challenges. CBF is a key indicator of cerebral function, providing a marker for cerebral wellbeing affecting daily life. In this work, we explored the impact of autonomic challenge on key indicator of brain health, through the combination of VM and blood flow measurement.

It is clear from this work that cerebral vascular control and skeletal muscle pH management are closely related, both at rest and after dynamic stimulation in CFS patients, indicating a strong influence of autonomic dysfunction on cerebral vascular control, However, further studies are required to fully appreciate the underlying pathology of CFS, especially CBF, oxygenation level measurement during VM and skeletal muscle perfusion measurement during rest and plantar flexion exercise.
